# Whole-Genome Sequence of the Cheese Isolate *Streptococcus macedonicus* 679

**DOI:** 10.1128/genomeA.01025-16

**Published:** 2016-09-22

**Authors:** Konstantinos Papadimitriou, Eleni Mavrogonatou, Alexander Bolotin, Effie Tsakalidou, Pierre Renault

**Affiliations:** a Department of Food Science and Human Nutrition, Laboratory of Dairy Research, Agricultural University of Athens, Athens, Greece; b Laboratory of Cell Proliferation and Ageing, Institute of Biosciences and Applications, National Centre for Scientific Research “Demokritos”, Athens, Greece; c Micalis Institute, INRA, AgroParisTech, Université Paris-Saclay, Jouy-en-Josas, France

## Abstract

It is well recognized that *Streptococcus macedonicus* can populate artisanal fermented foods, especially those of dairy origin. However, the safety of *S. macedonicus* remains to be established. Here, we present the whole-genome sequence of strain 679, which was isolated from a French uncooked semihard cheese made with cow milk.

## GENOME ANNOUNCEMENT

*Streptococcus macedonicus* has been identified as part of the fermenting flora of dairy foods around the world, and it presents some common traits to the well-established dairy starter *Streptococcus thermophilus* (1, 2). However, *S. macedonicus* belongs to the *Streptococcus bovis*/*Streptococcus equinus* complex, which includes species with a zoonotic potential that have been associated, among other conditions, with endocarditis, meningitis, and colon cancer (2, 3). Here, we present the genome sequence of *S. macedonicus* 679 isolated from French uncooked semihard cheese made with cow milk (4) that may facilitate the assessment of its safety when ingested with foods.

The genome of *S. macedonicus* 679 was sequenced by mate-pair Illumina sequencing (Mr DNA, Shallowater, TX). The library was prepared using the Nextera mate-pair sample prep kit (Illumina, San Diego, CA). Genome DNA (gDNA) was isolated and quantified. Subsequently, the sample underwent fragmentation, strand displacement, circularization, shearing, streptavidin purification, end repair, adenylation, and adapter ligation. The ligated adapters were utilized during a limited-cycle (10 cycles) PCR. Following the library preparation, the average library size was determined using the Agilent 2100 Bioanalyzer (Agilent Technologies, Palo Alto, CA). The library was sequenced by using the 600-cycle version 3 reagent kit (Illumina) in MiSeq (Illumina). Reads were assembled using SPAdes (5) against the published sequences of the chromosome and plasmid pSMA198 of *S. macedonicus* ACA-DC 198 as templates (6, 7). This reference-driven assembly resulted in two chromosomal contigs (contig_1, 79,307 bp; contig_2, 2,014,050 bp), as well as one plasmid contig (contig_3, 14,059 bp).

We employed an annotation transfer strategy using PANNOTATOR (8) and as reference the annotation of the *S. macedonicus* ACA-DC 198 genome. PANNOTATOR annotations were further enriched with RAST (9). The annotation transfer resulted in a total of 2,246 coding sequences (CDSs) in *S. macedonicus* 679 (84 in contig_1, 2,143 in contig_2, and 19 in contig_3). Among these CDSs, 75.1%, 7.7%, and 17.2% were 100%, 100 to 70%, and <70% identical to genes of *S. macedonicus* ACA-DC 198, respectively. We then inspected manually all annotation transfers of pseudogenes from strain ACA-DC 198 to strain 679. We identified 155 potential pseudogenes. This increased percentage of pseudogenes in the genome of *S. macedonicus* 679 is in agreement with previous observations for *S. macedonicus* ACA-DC 198, suggesting a genome decay evolution pattern that may be related to adaptation of the species to the rich in nutrients environment of milk (2, 6). Even though the majority of pseudogenes were common between the two strains, we could identify some that were present in one but not the other strain. This clearly indicates that genome decay took place early in the evolution of the species, but strain-specific selective pressures also generated distinct pseudogenes. The *S. macedonicus* 679 genome needs further investigation to better establish *in silico* its safety, its technological potential, and its adaptation traits to the dairy environment.

### Accession number(s).

The annotated genome sequence is available at the European Nucleotide Archive under the accession numbers FLZS01000001 to FLZS01000003.
